# Supplementary material for: Differences in Help-Seeking Behavior among University Students during the COVID-19 Pandemic Depending on Mental Health Status: Results from a Cross-Sectional Survey
Source: Behav Sci (Basel). 2023 Oct 25;13(11):885. doi: 10.3390/bs13110885 (PMC10669357; doi:10.3390/bs13110885)
Supplement: Supplementary file 1 [file behavsci-13-00885-s001.zip › behavsci-2648721-supplementary.pdf]

Supplementary Materials

Supplementary Table S1. Descriptives for Suicidal Ideation-Group (n = 1075)

	Mean	SD	Min.	Max.
Depressiveness (PHQ-9)	14.51	5.36	2	27
Anxiety (GAD-7)	11.95	4.76	0	21
Alcohol use (AUDIT-C)	2.96	2.52	0	11
Loneliness (UCLA)	6.92	1.79	3	9
Social Support (ESSI)	18.25	4.72	5	25
Stress (PSS-4)	9.82	2.92	0	16
Resilience (BRS)	2.59	0.74	1.00	5.00

Notes. SD, standard deviation; Min., minimum value; Max., maximum value; PHQ-9, Patient-Health-Questionnaire-9; GAD-7, Generalized Anxiety Disorder scale; AUDIT-C, Alcohol Use Disorder Identification Test; UCLA 3, 3-item Loneliness Scale; ESSI, ENRICHD Social Support Inventory; PSS-4, Perceived Stress Scale, BRS, Brief Resilience Scale.

Supplementary Table S2. Help-seeking intentions for Suicidal Ideation-Group (n = 1075)

	Mean	SD	Min.	Max.
Online services	2.11	1.10	1	4
Inpatient treatment	1.82	1.02	1	4
Psychological/psycho-social counselling	2.74	1.10	1	4
Psychotherapy	3.04	1.10	1	4
Psychiatrist	2.58	1.16	1	4

Notes. SD, standard deviation; Min., minimum value; Max., maximum value; PHQ-9, Patient-Health-Questionnaire-9; GAD-7, Generalized Anxiety Disorder scale; AUDIT-C, Alcohol Use Disorder Identification Test; UCLA 3, 3-item Loneliness Scale; ESSI, ENRICHD Social Support Inventory; PSS-4, Perceived Stress Scale, BRS, Brief Resilience Scale.

Supplementary Table S3. Descriptives for Symptoms-Group (n = 2,428)

	Mean	SD	Min.	Max.
Depressiveness (PHQ-9)	8.78	4.66	0	24
Anxiety (GAD-7)	8.49	4.54	0	21
Alcohol use (AUDIT-C)	3.91	2.36	0	12
Loneliness (UCLA)	5.73	1.83	3	9
Social Support (ESSI)	21.01	3.79	5	25
Stress (PSS-4)	7.53	2.98	0	16
Resilience (BRS)	2.98	0.75	1.00	5.00

Notes. SD, standard deviation; Min., minimum value; Max., maximum value; PHQ-9, Patient-Health-Questionnaire-9; GAD-7, Generalized Anxiety Disorder scale; AUDIT-C, Alcohol Use Disorder Identification Test; UCLA 3, 3-item Loneliness Scale; ESSI, ENRICHD Social Support Inventory; PSS-4, Perceived Stress Scale, BRS, Brief Resilience Scale.

.

Supplementary Table S4. Help-seeking intentions for Symptoms -Group (n = 2, 428)

	Mean	SD	Min.	Max.
Online services	2.19	1.11	1	4
Inpatient treatment	1.59	0.87	1	4
Psychological/psycho-social counselling	2.91	1.03	1	4
Psychotherapy	2.98	1.07	1	4
Psychiatrist	2.50	1.11	1	4

Notes. SD, standard deviation; Min., minimum value; Max., maximum value; PHQ-9, Patient-Health-Questionnaire-9; GAD-7, Generalized Anxiety Disorder scale; AUDIT-C, Alcohol Use Disorder Identification Test; UCLA 3, 3-item Loneliness Scale; ESSI, ENRICHD Social Support Inventory; PSS-4, Perceived Stress Scale, BRS, Brief Resilience Scale.

.

Supplementary Table S5. Descriptives for No-Symptoms-Group (n = 1,971)

	Mean	SD	Min.	Max.
Depressiveness (PHQ-9)	4.46	2.46	0	9
Anxiety (GAD-7)	4.57	2.36	0	9
Alcohol use (AUDIT-C)	1.66	1.31	0	4
Loneliness (UCLA)	5.14	1.63	3	9
Social Support (ESSI)	21.67	3.54	5	25
Stress (PSS-4)	5.46	2.43	0	15
Resilience (BRS)	3.35	0.68	1.33	5.00

Notes. SD, standard deviation; Min., minimum value; Max., maximum value; PHQ-9, Patient-Health-Questionnaire-9; GAD-7, Generalized Anxiety Disorder scale; AUDIT-C, Alcohol Use Disorder Identification Test; UCLA 3, 3-item Loneliness Scale; ESSI, ENRICHD Social Support Inventory; PSS-4, Perceived Stress Scale, BRS, Brief Resilience Scale.

Supplementary Table S6. Help-seeking intentions for No-Symptoms - Group (n = 1,971)

	Mean	SD	Min.	Max.
Online services	2.21	1.09	1	4
Inpatient treatment	1.63	0.88	1	4
Psychological/psycho-social counselling	2.90	1.00	1	4
Psychotherapy	2.91	1.05	1	4
Psychiatrist	2.44	1.09	1	4

Notes. SD, standard deviation; Min., minimum value; Max., maximum value; PHQ-9, Patient-Health-Questionnaire-9; GAD-7, Generalized Anxiety Disorder scale; AUDIT-C, Alcohol Use Disorder Identification Test; UCLA 3, 3-item Loneliness Scale; ESSI, ENRICHD Social Support Inventory; PSS-4, Perceived Stress Scale, BRS, Brief Resilience Scale.
